# Yields and costs of recruitment methods with participant phenotypic characteristics for a diabetes prevention research study in an underrepresented pediatric population

**DOI:** 10.1186/s13063-020-04658-8

**Published:** 2020-08-14

**Authors:** Kiley B. Vander Wyst, Micah L. Olson, Elva Hooker, Erica G. Soltero, Yolando P. Konopken, Colleen S. Keller, Felipe G. Castro, Allison N. Williams, Arlene D. R. Fernández, Donald L. Patrick, Stephanie L. Ayers, Houchun H. Hu, Armando Peña, Janiel Pimentel, William C. Knowler, Gabriel Q. Shaibi

**Affiliations:** 1 Center for Health Promotion and Disease Prevention, Edson College of Nursing and Health Innovation, Arizona State University, Phoenix, AZ USA; 2 Division of Pediatric Endocrinology and Diabetes, Phoenix Children’s Hospital, Phoenix, AZ USA; 3 Ivy Center for Family Wellness, The Society of St. Vincent de Paul of Arizona, Phoenix, AZ USA; 4 USDA/ARS Children’s Research Center, Baylor College of Medicine, Houston, TX USA; 5 Department of Health Services, University of Washington, Seattle, WA USA; 6 Southwest Interdisciplinary Research Center, Arizona State University, Phoenix, AZ USA; 7 Hyperfine Research, Inc., Guilford, CT USA; 8 Diabetes Epidemiology and Clinical Research Section, National Institute of Diabetes and Digestive and Kidney Diseases, Phoenix, AZ USA

## Abstract

**Background/aims:**

Prediabetes and diabetes disproportionately impact Latino youth, yet few diabetes prevention programs have prioritized inclusion of this underrepresented population. This report describes the recruitment process, yields, associated costs, and phenotypic characteristics of Latino youth with obesity and prediabetes enrolled in a randomized controlled diabetes prevention study in the USA.

**Methods:**

Recruitment efforts included referrals from clinics, community outlets, local media, and word of mouth with the goal of enrolling 120 Latino adolescents aged 12–16 with obesity (BMI > 95th percentile) and prediabetes. Prediabetes eligibility was determined by any of the following: HbA1c between 5.7 and 6.5%, fasting glucose between 100 and 125 mg/dL, or a 2-h glucose between 120 and 199 mg/dL following a 75-g oral glucose tolerance test (OGTT), but not meeting any of the diagnostic criteria for diabetes. Eligible participants were randomized 2:1 to either a 6-month community-based lifestyle intervention that included group nutrition and health education classes (1 day/week) and group exercise classes (2 days/week) or usual care control arm. Recruitment yields were determined by review of referral source in the study screening database. Recruitment costs were determined by an after-the-fact financial review of actual and in-kind costs. Participant phenotypic characteristics (i.e., demographics, anthropometrics, and biochemical data) were compared by recruitment strategy using a one-way ANOVA.

**Results:**

Recruitment efforts covered 160 mile^2^ (414 km^2^) across 26 ZIP codes (postcode) in the Phoenix Metropolitan Area and yielded 655 referrals from clinics (*n* = 344), community (*n* = 143), media (*n* = 137), and word-of-mouth (*n* = 31). From this pool, 26% (*n* = 167) did not meet general, pre-screening eligibility criteria; 29% (*n* = 187) declined participation; and 10% (*n* = 64) were unable to be contacted. A total of 237 youth were invited to the clinical research unit to determine final eligibility. Following the OGTT, 52% (*n* = 122) met prediabetes criteria and 117 were subsequently randomized. Clinical recruitment yielded the highest number of referrals (53%; *n* = 344) while word-of-mouth yielded the highest proportion (35%; *n* = 11) of randomized participants per referred youth. There were no significant differences in anthropometric or biochemical measures among youth by recruitment strategy. Based upon final enrollment numbers, community recruitment was the costliest approach ($486/randomized participant) followed by clinical ($248/randomized participant) and media ($236/randomized participant).

**Conclusions:**

The ability to meet enrollment goals for a clinical trial of an underrepresented population required multiple recruitment strategies. Although strategies vary in yields and costs, it appears they produce similar phenotypical risk profiles of eligible youth.

**Trial registration:**

ClinicalTrials.gov NCT02615353. Registered on 26 November 2015

## Background

In the USA, 1 in 5 adolescents has prediabetes [1] which puts them at increased risk for the development of type 2 diabetes (T2D) and cardiovascular disease [2]. Prediabetes disproportionately affects Latino adolescents (22.7%) compared to non-Hispanic white youth (15.8%) [1] and is characterized by impaired glucose regulation and tolerance [3]. Despite increased awareness of prediabetes in the pediatric population, few clinical trials have prioritized the inclusion and enrollment of this high-risk sub-group in their designs [4]. A contributing factor to the limited number of trials focused on youth with prediabetes is the issue of identifying and categorizing prediabetes in the pediatric population [5, 6] which may pose challenges for recruitment.

Although recruitment strategies vary based on the study, disease outcome, and population of interest, it is unclear if the use of different recruitment strategies within a single study influences the phenotypic characteristics of the sample. A previous pilot study focused on recruiting high-risk minority youth for a diabetes prevention program reported that clinical referrals yielded a greater proportion of youth with prediabetes compared to those referred through community channels [7]. However, the absolute number of youth referred through the community (*n* = 156) was much higher than those received from clinical sites (*n* = 30). Strategies that span multiple referral sources may result in a more representative sample and, as a result, contribute to heterogeneity in demographic, anthropometric, and biochemical data by referral source [8, 9]. Since clinical trials test the efficacy of interventions, it is vital to examine the challenging but often overlooked recruitment and screening processes and whether recruitment strategies influence the phenotypic characteristics of the sample. Moreover, how various strategies contribute to trial costs particularly for underserved and underrepresented populations is an area in need of further research [10].

The primary recruitment goal of clinical trials is to meet a priori sample sizes, with less attention given to how the target sample is achieved. Numerous papers describe recruitment barriers and potential alternative strategies [11–14], which are amplified among underrepresented populations including minorities [11, 15, 16] and children [15, 16]. Previous research suggests that combining community-based and mass-media outreach strategies while incorporating cultural values and linguistically appropriate materials are successful strategies for recruiting Latino youth into clinical trials [16]. These clinical trials also report that involvement of community leaders [16], providing monetary incentives for participation [16, 17], and accommodating parent/caregiver schedules [17] resulted in the successful recruitment of Latino youth. However, recruitment of high-risk populations such as those with prediabetes remains challenging because risk assessment is often identified clinically and may be missed in trials that rely exclusively on community efforts. Therefore, recruitment efforts that span systems (i.e., clinical, community, and academic) and strategies (e.g., media, technology, marketing, and incentives) will likely yield the highest number of participants [16, 18, 19]. Expanding recruitment reports for clinical trials to include both internal and external aspects will better inform future dissemination and implementation efforts of successful interventions [20].

As funding organizations continue to prioritize the importance of clinical trial research and health disparities continue to widen, the need for more comprehensive reporting as well as understanding the processes and costs for enrolling underrepresented populations is warranted. Therefore, the objectives of this report are to (1) describe screening and recruitment processes, (2) evaluate recruitment yields by strategy, (3) report recruitment and screening costs, and (4) evaluate phenotypic characteristics by referral strategy of Latino youth with prediabetes enrolled in a randomized controlled lifestyle intervention. Additionally, we offer lessons learned throughout the process.

## Methods

### Study design and participants

Details of the study design and methods can be found elsewhere [21]. Briefly, the randomized controlled trial tests the efficacy of a culturally grounded, community-based lifestyle intervention as compared to usual care control (UCC) on changes in T2D risk factors. The six-month lifestyle intervention includes nutrition and health education, physical activity, and behavior change strategies. Participants and families participated in group nutrition and health education classes (1 day/week for 20 weeks) and youth attended group exercise classes (3 days/week for 60 min for 20 weeks). Participants randomized to the UCC met with a pediatric endocrinologist and a registered dietitian to review laboratory results and receive lifestyle counseling at baseline and 6 months. The study recruitment and enrollment plan called for equal numbers of males and females meeting the following inclusion criteria: (1) self-identified as Latino; (2) aged 12–16 years old at enrollment; (3) obese, defined as a BMI ≥ 95th percentile for age and sex or a BMI ≥ 30 kg/m^2^; and (4) prediabetic, defined as an HbA1c between 5.7 and 6.5%, fasting glucose between 100 and 125 mg/dL, or 2-h glucose between 120 and 199 mg/dL, but not meeting any of the diagnostic criteria for diabetes. These inclusion criteria for prediabetes are in line with the American Diabetes Association diagnostic criteria for T2D [22], with the exception of post prandial glucose levels. The expanded definition for post prandial glucose was used as previous studies have shown that youth with 2-h glucose of > 120 mg/dL have similar diabetes risk and rates of conversion to T2D as youth with 2-h glucose > 140 mg/dL [23]. Recruitment for the trial began May 2016 and ended September 2019.

### Recruitment methods

Given the narrowly defined eligibility criteria to enroll Latino youth with prediabetes, it was necessary for key study personnel to span multiple institutions (Supplemental Figure 1) and utilize a combination of recruitment methods that included clinical, community, media, and word-of-mouth strategies. Each method and associated strategy are outlined below.

#### Clinical strategy

Local ambulatory pediatric clinics (*n* = 21) were identified from the network of clinics that refer patients to our clinical partners at Phoenix Children’s Hospital and were located within a 15-mile (24 km) radius from the intervention delivery sites. These clinics were contacted through letters and emails from the project physician that provided a brief description of the trial. It is also worth noting the majority of clinical referrals were from federally qualified health centers or specialty clinics who serve a large proportion of Latino patients. Once interest was established, the study program manager contacted the clinic manager to discuss the purpose of the study, the logistics of being a recruitment referral site, and to schedule an in-person presentation. The project physician made 22 presentations to clinic staff about the trial including information about prediabetes and T2D in children. Clinic-specific patient referral procedures were developed in consultation with clinic staff and coordinated by the research program manager. Referral procedures included a variety of staff (e.g., physician, nurse, registered dietitian, medical assistant) who discussed the trial and provided a study flyer to potentially eligible patients and families during clinic visits. Interested individuals signed a “Release of Information” form that was faxed to the research team who would subsequently contact potential participants to initiate the eligibility screening process. As a result of consistent and close communication with one clinic, an automatic notification system was built into the electronic health record system that would flag study eligible patients and prompt the provider to discuss the study at the next clinic visit.

Monthly reports were generated for each clinic that included the total number of referred patients that were eligible, enrolled, or declined participation. In addition to these reports, laboratory results generated over the course of the research study were faxed back to the referring clinic manager. Given that recruitment occurred over a period of 40 months, research staff periodically returned to the clinics to provide study updates and solicit feedback on referral mechanisms approximately every six months. Additionally, participants’ laboratory information was returned immediately to the referring clinic.

#### Community strategy

Community recruitment efforts were led by our partners from the St. Vincent de Paul (SVdP) Ivy Center for Family Wellness. The SVdP Ivy Center for Family Wellness was established in 2000 with a goal of building healthier communities through awareness, knowledge, and empowerment of individuals and families. SVdP emphasizes prevention and management of T2D by delivering culturally grounded health education programs for high-risk populations. Staff solicited recruitment presentation opportunities through their network of schools, school-based health clinics, churches, and community health coalitions. In addition, representation at community health fairs and local swap meets provided an opportunity to directly interface with potential participants and their families. A member of the SVdP team contacted the community event coordinator, provided a brief description of the trial, and expressed interest in recruiting from the event. Representatives from SVdP staffed community events with educational handouts, study flyers, incentives (e.g., toothbrushes, pillbox organizers, toys, and food storage containers), and raffles to increase engagement. Interested individuals provided their contact information to the SVdP staff member who put them in contact with the ASU research staff to receive more information about the study, answer any additional questions, and begin the screening process.

#### Media

Study staff targeted a variety of Spanish-language media outlets including advertisements in electronic books, magazines, local newspapers, and social media (i.e., Instagram® and Facebook®). A strategic partnership was created with Segunda Mano Magazine, a local magazine for the Latino community in Phoenix, AZ, with a weekly distribution reaching 370,000 people through the printed and electronic magazine. As part of this partnership, the magazine included a weekly study advertisement (Supplemental Figure 2) in exchange for SVdP staff writing health-related articles (see Supplemental Figure 3 for an example). The trial was also advertised on four local radio stations that serve the Latino community. Social media recruitment included posting study flyers and recruitment materials on Facebook® pages and Instagram® accounts that highlighted the potential benefits of participation (e.g., incentives, health screening, nutrition and physical activity education, and free YMCA membership) and contained study staff contact information.

#### Word-of-mouth strategies

Word-of-mouth recruitment included referrals from participants and/or families of the current trial and other studies from our research team that focused on diabetes in Latino youth within the Phoenix Metropolitan Area.

### Screening process

The study employed a three-tiered screening and enrollment process that included a pre-screen phone interview with parents and youth, then an in-person consent and assent visit followed by an in-person health screening visit to determine final eligibility. The first step of the screening process involved SVdP and research staff contacting potential participants to complete a pre-screen phone interview. This pre-screen phone interview confirmed the age, obesity status by estimating BMI via parent report of height and weight, and ethnicity of the potential participant. Additionally, research staff confirmed that the youth was able to participate in physical activity without limitations, had English literacy, was able to participate in intervention classes and booster sessions, and was not taking any medications or diagnosed with a condition that was exclusionary. Parents verified that the family had no plans to relocate within the next year. If youth were eligible after pre-screening and both the parents and youth remained interested in participation, families were scheduled to come in for consenting and a health screening visit at the ASU Clinical Research Unit.

The in-person consent/assent process occurred prior to but on the same day as the health screening visit. During the consent, bilingual/bicultural research staff explained the study in full detail to the parent and youth, including the procedures for the health screening, randomization process, participation requirements for intervention and UCC groups, risks and potential benefits from participation, and answered any questions.

For participants who consented, final eligibility was determined through a health screening that included height and weight used to calculate and confirm BMI percentile as well as a standard 75-g oral glucose tolerance test (OGTT) to determine prediabetes status. Blood specimens collected during the OGTT were sent to a CLIA-approved clinical laboratory to determine HbA1c (Roche Cobas® C513), and fasting and 2-h glucose concentrations (Roche Cobas® 8000). After completion of the health screening visit, youth were compensated $40 and the parent/guardian was provided $10 for parking. Families (youth and parent/guardian) were contacted within seven days by phone once final eligibility was confirmed. If the youth were deemed eligible, baseline testing was scheduled within four weeks of the health screening visit while those that were ineligible were sent a letter from the principal investigator briefly describing their results and the suggestion to share the results with their primary care provider (Supplemental Figure 4).

### Statistical analyses

Referral yields are summarized as counts and percentages by strategy (i.e., clinic, community, media, word-of-mouth). Recruitment costs were determined by an after-the-fact financial review of actual and in-kind costs associated with the various strategies, which included staff, community event attendance costs, supplies (e.g., flyers and incentives), food for clinic staff during recruitment presentations, and mileage reimbursement. Baseline characteristics were compared by recruitment strategy using one-way ANOVA. All analyses were performed in SPSS version 25 with significance deemed at the *P* < 0.05.

## Results

### Recruitment

**Fig. 1:**
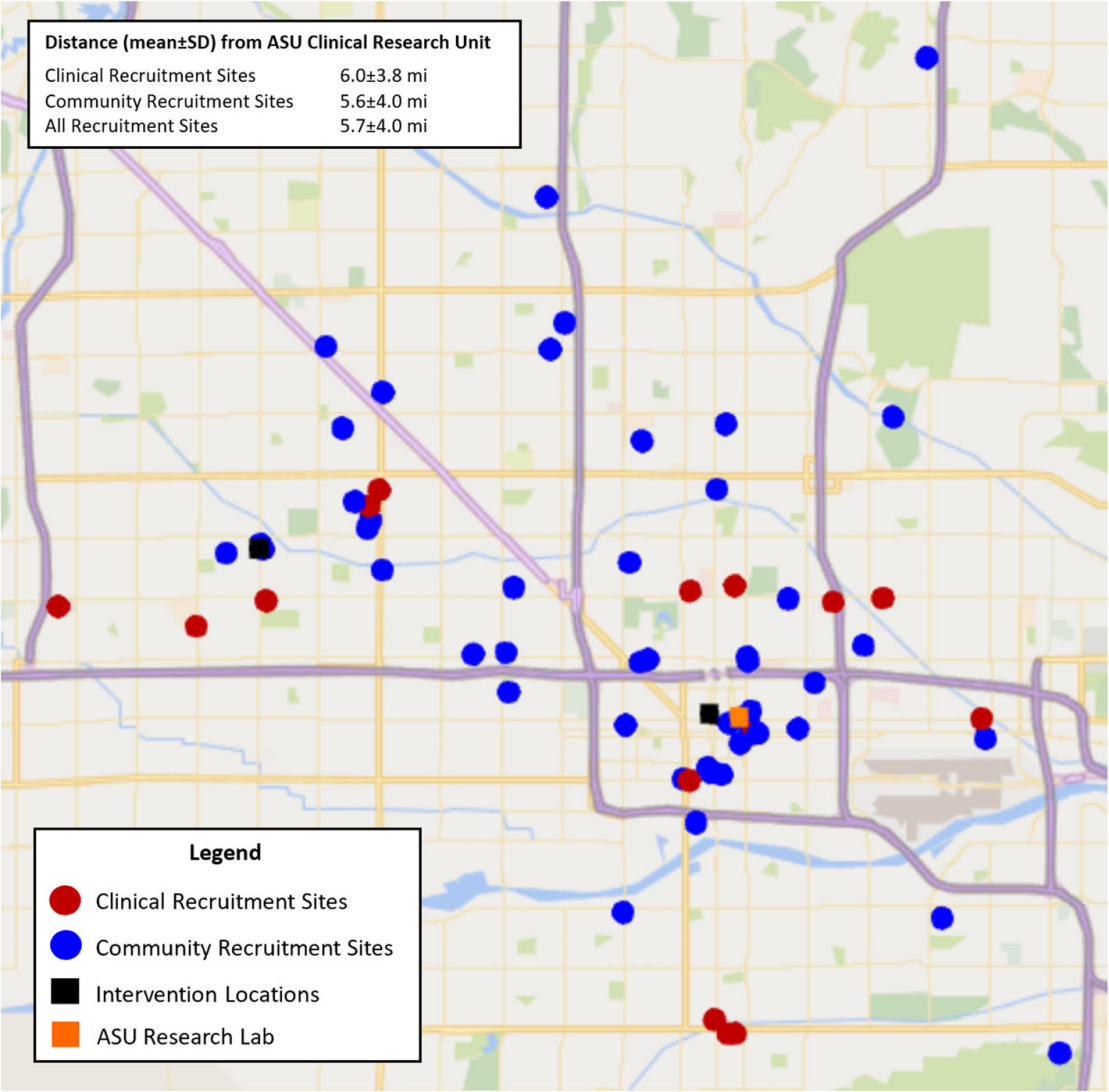
Reach of community and clinical recruitment efforts

Recruitment efforts covered 26 ZIP codes (postcodes) across 160 mile^2^ (414 km^2^) of the Phoenix Metropolitan Area (Fig. 1). The average (± SD) distance from the recruitment site to the clinical research unit was 5.7 ± 4.0 miles with a range of 0.3 to 14.1 miles. Clinical recruitment sites (6.0 ± 3.8 miles) were marginally farther than community recruitment locations (5.6 ± 4.0 miles, *P* = 0.73).

**Fig. 2:**
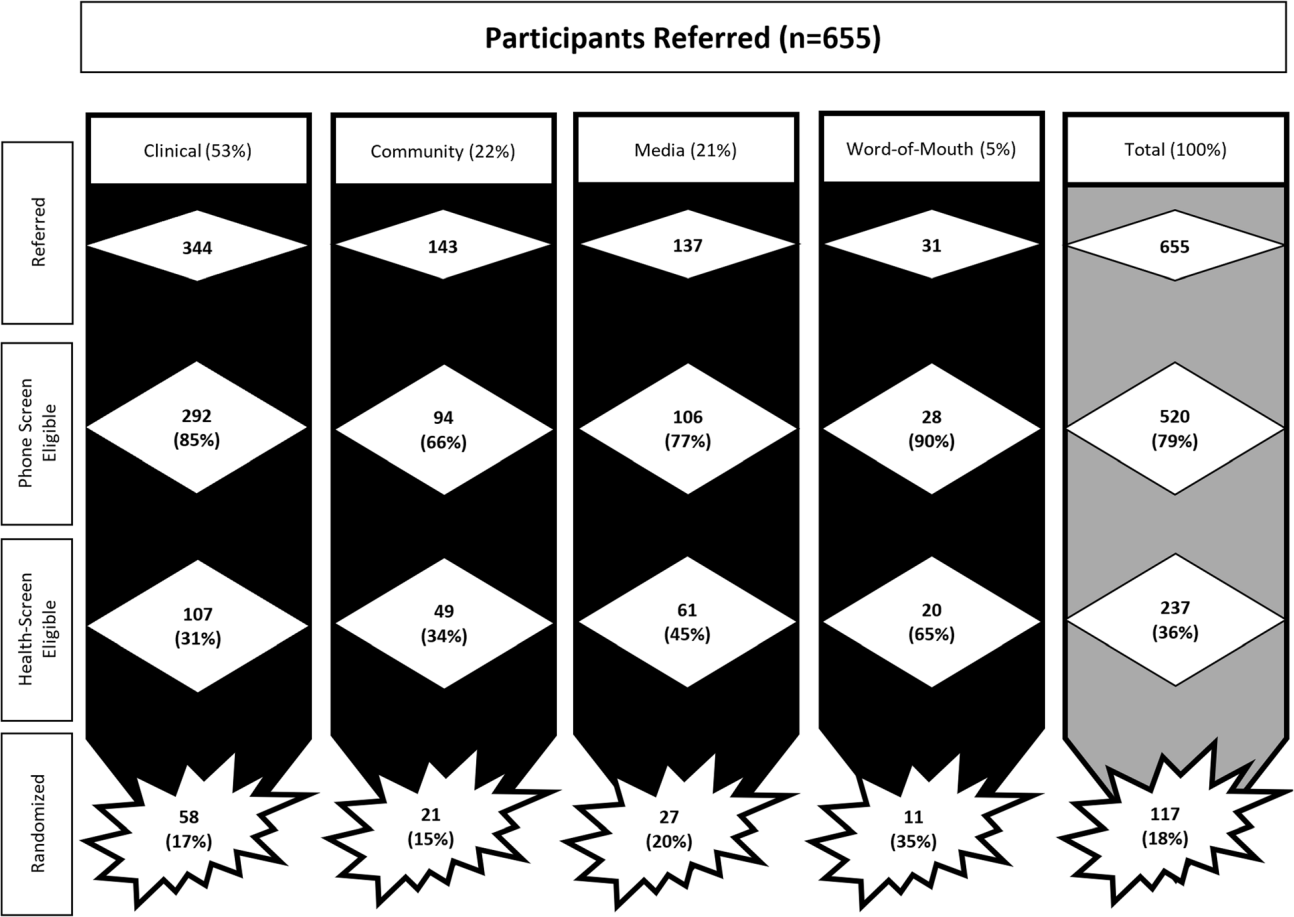
Recruitment, screening, and randomization flow diagram

In total, 655 youth were referred to the study with 520 (79.4%) pre-screened by phone, 237 (36.2%) completing laboratory screening, and 122 (18.6%) meeting eligibility criteria. Clinical recruitment strategies yielded the highest number of referred youth (*n* = 344) but resulted in the second lowest proportion of randomized participants (17%). Word-of-mouth recruitment efforts yielded the lowest number of referrals but the highest proportion of randomized youth (31%). Community and media recruitment strategies yielded similar total referrals (143 vs 137, respectively) with community efforts having a slightly lower proportion of randomized participants (15%) compared to media (20%). Figure 2 provides an overview of the referred youth by recruitment strategy along with numbers and proportions of participants at each level of the screening processes. Although 122 were deemed eligible, five of these youth declined enrollment after the health screening visit but prior to randomization resulting in a final sample of 117 participants with 12.8% (*n* = 15) of youth withdrawing after randomization (Supplemental Table 1). In total, 243 (37.1%) youth declined participation in the study due to lack of time (11.1%), lack of interest (5.8%), or distance (8.2%) with the remaining (74.9) unknown/not specified.

### Phenotypic characteristics

Table 1 summarizes the characteristics of participants who completed the health screening visit by recruitment strategy. Clinical recruitment sources referred a significantly lower percentage of males (55.1%) compared to all other recruitment sources (70.8% community, media, and word-of-mouth combined, *P* = 0.02). There were no other differences in demographic (age, parental T2D history, or gestational diabetes history), anthropometric (height, weight, BMI, or BMI percentile), or glycemic indicators (2-h glucose, fasting glucose, HbA1c) among screened (Table 1) or randomized (Supplemental Table 2) youth by recruitment source.

**Table 1:** Characteristics of Latino youth who completed health screening (*N* = 237) in a diabetes prevention program by recruitment site

Source	Clinical	Community	Media	Word-of-mouth	***P*** value
	*N* = 107	*N* = 49	*N* = 61	*N* = 20	
Age, years	13.9 ± 0.1	14.2 ± 0.2	13.6 ± 0.2	13.7 ± 0.3	0.17
Sex, % male (*N*)	55.1% (59)	77.6% (38)	62.3% (38)	80.0% (16)	**0.02**
Height, cm	163.4 ± 0.8	165.2 ± 1.2	162.2 ± 1.0	163.2 ± 3.9	0.41
Weight, kg	90.6 ± 1.8	88.7 ± 2.7	87.5 ± 2.3	99.2 ± 5.6	0.12
BMI, kg/m^2^	33.6 ± 0.5	32.4 ± 0.8	33.0 ± 0.7	34.4 ± 1.1	0.40
BMI percentile, %	98.1 ± 0.2	97.5 ± 0.4	98.1 ± 0.2	98.7 ± 0.2	0.06
Glucose					
Fasting, mg/dL	89.0 ± 0.2	85.5 ± 1.9	91.2 ± 3.7	89.5 ± 1.9	0.30
2-h, mg/dL	119.1 ± 2.7	109.6 ± 4.0	116.3 ± 6.6	117.6 ± 4.3	0.47
HbA1c, %	5.5 ± 0.03	5.6 ± 0.04	5.6 ± 0.12	5.5 ± 0.07	0.99
Parental T2D history, % yes (*N*)	29.9% (32)	13.5% (8)	13.1% (8)	20.0% (4)	0.05
Gestational diabetes mellitus history, % yes (*N*)	55.2% (32)	17.2% (10)	24.1% (14)	3.4% (2)	0.15

Data are presented as estimated marginal means and standard error for continuous variables and percentages (*N*) for categorical variables. Univariate general linear models were performed for group comparisons for continuous variables and chi-square test statistics were performed to compare group differences for categorical variables. Significance was determined at *P* = 0.05

### Costs

Total costs to recruit, screen, and enroll the 117 participants were estimated at $113,560.12. Table 2 provides the recruitment and screening costs per randomized participant by recruitment strategy. Screening costs were $752/randomized participant and included research and laboratory staff costs, participant compensation, and supplies. Community recruitment was the costliest approach ($486/randomized participant) followed by clinical ($248/randomized participant) and then media ($236/randomized participant). Media recruitment was subsidized through a partnership between our collaborators from the SVdP Ivy Center for Family Wellness and Segundo Mano where typical costs for magazine advertisements ($250 per ad space) were exchanged for writing health-related articles (Supplemental Figure 3). This arrangement resulted in a savings of more than $46,000 or approximately $1911.70/enrolled participant.

**Table 2:** Screening and recruitment costs per participant enrolled

	Number of units	Cost	Total
**Screening costs**			
Recruitment flyer (research coordinator hours)	20	$ 23.00	$ 460.00
Eligibility phone screening (research technician hours)	1.25	$ 17.50	$ 21.88
Health screening			
Reminder calls/text messages	0.25	$ 17.50	$ 4.38
Staff (visit preparation, consent, data collection, and cleanup)	6.08	$ 17.50	$ 106.40
Visit (supplies, phlebotomy, labs)	1	$ 100.00	$ 100.00
Participant compensation			
Incentive	1	$ 40.00	$ 40.00
Parking	1	$ 10.00	$ 10.00
Laboratory interpretation			
Physician professional fee	0.005	$ 90.62	$ 0.45
Data entry	0.6	$ 14.65	$ 8.79
**Screening cost total per participant**			**$ 751.89**
**Recruitment costs**			
***Clinical***			
Monthly communication with practices	15	$ 17.50	$ 262.50
Lunch and learn			
Schedule and prepare (hours)	33	$ 53.89	$ 1778.37
Principal investigator time (hours)	16	$ 117.69	$ 1883.04
Program manager time (hours)	44	$ 53.89	$ 2371.16
Project physician/co-investigator time (hours)	44	$ 104.19	$ 4584.36
Lunch	22	$ 154.01	$ 3388.22
Mileage	220	$ 0.55	$ 121.00
**Clinical recruitment total per participant (*n*** **= 58)**			**$ 248.08**
**Community**			
Exhibitor/vender fee (average $350/table)	10	$ 350.00	$ 3500.00
Exhibitor/vender staffing hours	335	$ 18.00	$ 6030.00
Mileage (Avg. 15 miles per event)	1260	$ 0.54	$ 680.40
**Community recruitment total per participant (*n*** **= 21)**			**$ 486.21**
**Media**			
Magazine advertisement development staff hours	312	$ 18.00	$ 5616.00
Interview (radio, TV, PSA) staff hours	29	$ 18.00	$ 522.00
Mileage (Avg. 15 miles per event)	435	$ 0.54	$ 234.90
**Media recruitment total per participant (*n*** **= 27)**			**$ 236.03**

## Discussion

Randomized clinical trials (RCTs) are often the preferred approach to evaluating therapeutic interventions. Successful RCTs seldom report detailed information on the resources, personnel, infrastructure, and costs required to meet target enrollment numbers. In the current study, we found that clinical recruitment yielded the highest number of referrals but was the second most costly approach, community recruitment had the second highest number of referrals but was the costliest approach, and media recruitment efforts yielded the lowest number of referrals but was the most cost efficient. Additionally, word-of-mouth resulted in the lowest number of total referrals but yielded the highest proportion of randomized participants per referred youth. Despite differences in recruitment yields by strategy, there were no significant differences in anthropometric or biochemical measures among youth by recruitment strategy. This study demonstrated that in order to meet the enrollment goal of a community-based, randomized controlled trial that prioritized an underrepresented population, multiple recruitment strategies across diverse settings were required.

Although the majority of NIH-funded clinical trials are housed in academic health centers, the development of General Clinical Research Centers (GCRCs) and Clinical and Translational Science Award (CTSA) has expedited the translation of basic and clinical research into clinical and community practice [24]. Both programs provide research infrastructure support for collaborative, multi-disciplinary teams but more importantly foster rapid dissemination of research findings [24]. With the assumption that diabetes prevention is most effective in the early stages of the pathogenesis [23] and will require interventions that extend beyond the clinic [25], the development and fostering of multi-sector strategic partnerships is vital for translating evidenced-based health promotion and diabetes prevention programs. While cost and participant yields differed by strategy, there were no differences in participant phenotypic characteristics by recruitment source.

Lack of consensus regarding the optimal recruitment method results in the majority of clinical trials employing multiple strategies in order to meet enrollment numbers. Recruitment efforts from local pediatric clinics led to the greatest number of referrals which is similar to another youth pilot diabetes prevention program [7]. The current study provided monthly laboratory reports and patient health screening results to referring pediatric clinics in an effort to optimize communication and increase recruitment referrals. However, word-of-mouth recruitment strategies led to the highest yield of randomized participants per referred youth. This is similar to other studies which found that passive and snowball recruitment efforts resulted in the largest yield of eligible participants [8, 9, 26]. The success of word-of-mouth recruitment may be due to the natural trust that comes with recommendations from family or friends; therefore, minimizing the fear, anxiety, or stress associated with participating in research studies [27]. The current study also utilized culturally relevant recruitment materials which have been shown to increase the proportion of participants that schedule a baseline screening visit [28]. Furthermore, the current study also provided financial incentives for completion of the health screening visit which has previously been shown to increase the recruitment of research participants [14, 29], particularly among youth into T2D clinical trials [19]. The different recruitment yields by strategy demonstrated the need for clinical trials to use a variety of methods in order to reach a greater pediatric population at risk of developing T2D.

Although recruitment strategies vary based on the study, disease outcome, and population of interest, it is unclear if the use of different strategies leads to differences in participants that ultimately enroll. It could be argued that recruitment strategies that reach across multiple referral sources result in a more representative sample and even more so when prioritizing vulnerable and underrepresented populations that experience disparities in access to care [30] and research opportunities [17, 31]. Unlike other studies [8, 9], the current study did not find any differences in demographics, anthropometrics, or biochemical data by recruitment strategy. It is important that clinical trials evaluate not only the success of various recruitment efforts but also how recruitment strategies influence the phenotypic characteristics of the sample.

Despite efforts to better understand reasons that affect the participation and retention of hard-to-reach populations in clinical trials [9, 16, 19, 32–34], previous research has shown that there are large numbers of eligible individuals that decline participation [35]. The current study had 37% of participants decline participation which was lower than the 51% of Diabetes Prevention Program (DPP) participants who declined for unknown reasons or lack of interest [35]. In the current study, the main reasons for declining participation were the lack of time, distance, or interest which is similar to other reports that identified inconvenience as one of the most prevalent reasons for declining participation [36]. Previous literature has reported that participation could be enhanced through annual evaluation of recruitment refusal data and modification of procedures to accommodate the needs of potential participants [36]. Although the current study did not begin evaluating reasons for declining participation until halfway through the study, several other strategies were employed to increase participation including constant communication with families, flexibility in scheduling screening visits, and providing transportation compensation to and from health screening visits. Despite these efforts, the current study had a large proportion of participants and their families decline participation. It is important for future studies to understand the reasons why eligible participants decline enrollment into clinical trials to aid in the dissemination and implementation of effective evidenced-based interventions.

Despite the high cost associated with conducting a clinical trial [37], there is a large variation in the reported costs of recruitment, ranging from $18 to over $1000 per participant [9, 26, 35]. The DPP reported that the average cost per randomized participant equated to $1075 [35] but did not breakdown cost by recruitment strategy. The current study found that the media recruitment strategy was the most cost effective followed by clinical and then community recruitment efforts. The community recruitment efforts were most cost prohibitive due to the time, personnel, and resources needed to attend community events. This is unlike previous reports that found proactive recruitment (i.e., healthcare provider or research staff) cost more per enrolled participant than reactive methods such as flyers and media [9, 26]. In contrast, the estimated annual healthcare costs associated with treating both type 1 and 2 diabetes among youth (aged < 18 years old) were $7510 per person in 2017 [38]. In 2012, the average lifetime medical costs for treating adults (aged > 25 years old) with type 2 diabetes were estimated to be $85,200 [39]. Regardless of the cost difference, recruitment and screening processes cost clinical trials a substantial amount of resources and personnel yet there is little consensus on the most cost effective approach. Therefore, it is vital that researchers develop a systematic method of tracking and reporting associated costs for recruitment to clinical trials to inform future studies.

Lessons learned include the importance of real-time, detailed documentation of all components of the recruitment process, efficient and clear communication, and establishment and maintenance of relationships with participants, their families, and external partners. The importance of documenting multiple recruitment calls, detailed expense reporting, and recruitment yields by strategy became apparent while the study was in progress. Accurately tracking this information in an easily accessible database allowed for timely evaluation of efforts and modifications to resources and personnel allocation. Detailed documentation of the actual time and cost required for each recruitment strategy also allowed for more transparent reporting to the scientific community. Given the number of individuals involved in the study that spanned multiple institutions under the same research program with several ongoing research studies, it was vital for the team to establish clear, effective communication at the onset of this study. This included weekly team meetings to track study metrics, use of project management software, identification of clinical and community recruitment champions, and creation of personnel and organizational flow charts. These modifications streamlined the communication by clearly delegating responsibilities, goals, and deadlines to the appropriate person and organization. Lastly, the relationships fostered with youth and their families as well as community and clinical partners who served as advocates for recruitment efforts are critical. Establishing rapport early in the process to ensure that youth and families are not perceived as “subjects” in an experiment but rather are participants that contribute as much to advancing science as anyone else involved in the research has proven to be key for continued engagement and growth of the research program. In addition, the research team has been engaged in health promotion and diabetes prevention in this community for over 10 years. This has resulted in an established network that has a history in the community that lends itself to a multi-sector, collaborative team for study implementation. The current study represents these longstanding relationships between the research team and our external partners.

## Conclusion

In conclusion, the success of meeting enrollment goals was dependent on the collaborative effort of a multi-disciplinary team that spanned several organizations and utilized various recruitment strategies. Although recruitment strategies differed in terms of participant yields and costs, phenotypic characteristics did not vary by recruitment strategy.

## Supplementary information

**Additional file 1: Supplemental Figure 1.** Network Connection Map.

**Additional file 2: Supplemental Figure 2.** Segunda Mano Magazine Study Advertisement.

**Additional file 3: Supplemental Figure 3.** Example of Segunda Mano Magazine Health Lifestyle-based Article.

**Additional file 4: Supplemental Figure 4.** Example Letter Provided to Parent/Guardian of Ineligible Youth.

**Additional file 5: Supplemental Table 1.** Participant Reasons for Withdrawing After Randomization.

**Additional file 6: Supplemental Table 2.** Baseline Characteristics of Latino Youth Randomized (*N* = 117) to a Diabetes Prevention Program by Recruitment Site.

## Data Availability

The datasets used and/or analyzed during the current study are available from the corresponding author on reasonable request.
