# Supplementary figures and images for: Yields and costs of recruitment methods with participant phenotypic characteristics for a diabetes prevention research study in an underrepresented pediatric population
Source: Trials. 2020 Aug 14;21:716. doi: 10.1186/s13063-020-04658-8 (PMC7429699; doi:10.1186/s13063-020-04658-8)

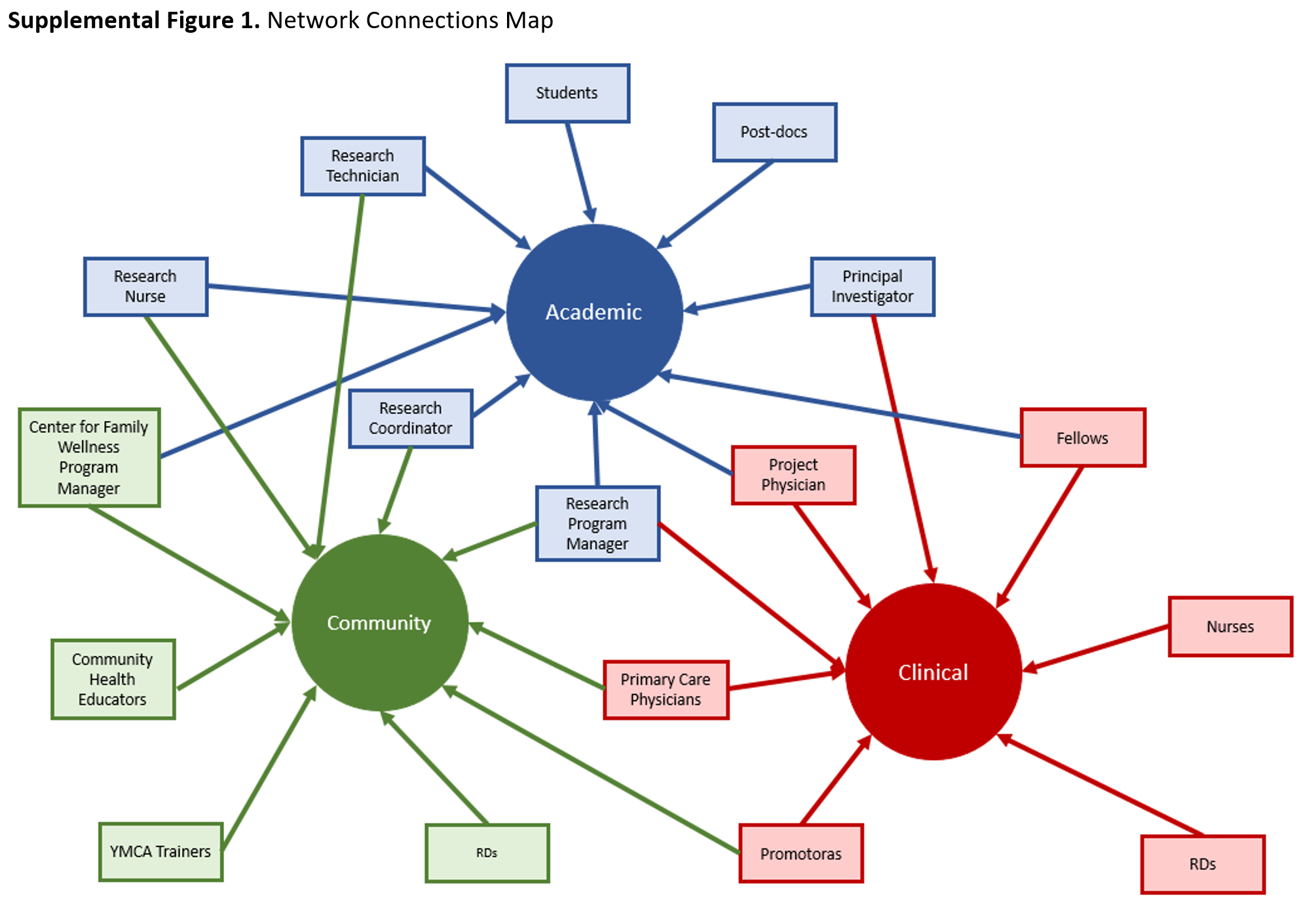

Supplement: Supplementary file 1 — Additional file 1: Supplemental Figure 1. Network Connection Map. [file 13063_2020_4658_MOESM1_ESM.png]

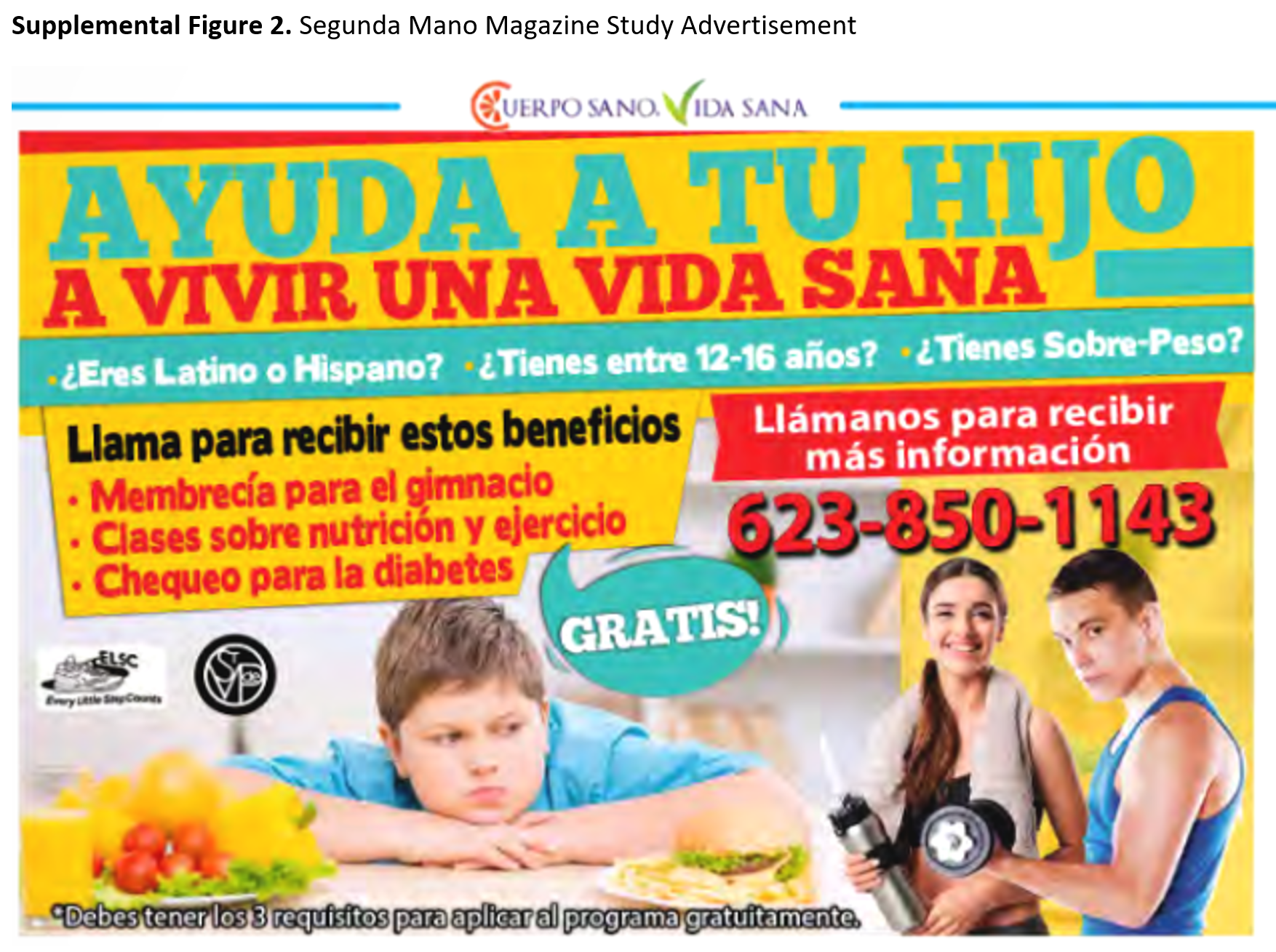

Supplement: Supplementary file 2 — Additional file 2: Supplemental Figure 2. Segunda Mano Magazine Study Advertisement. [file 13063_2020_4658_MOESM2_ESM.png]

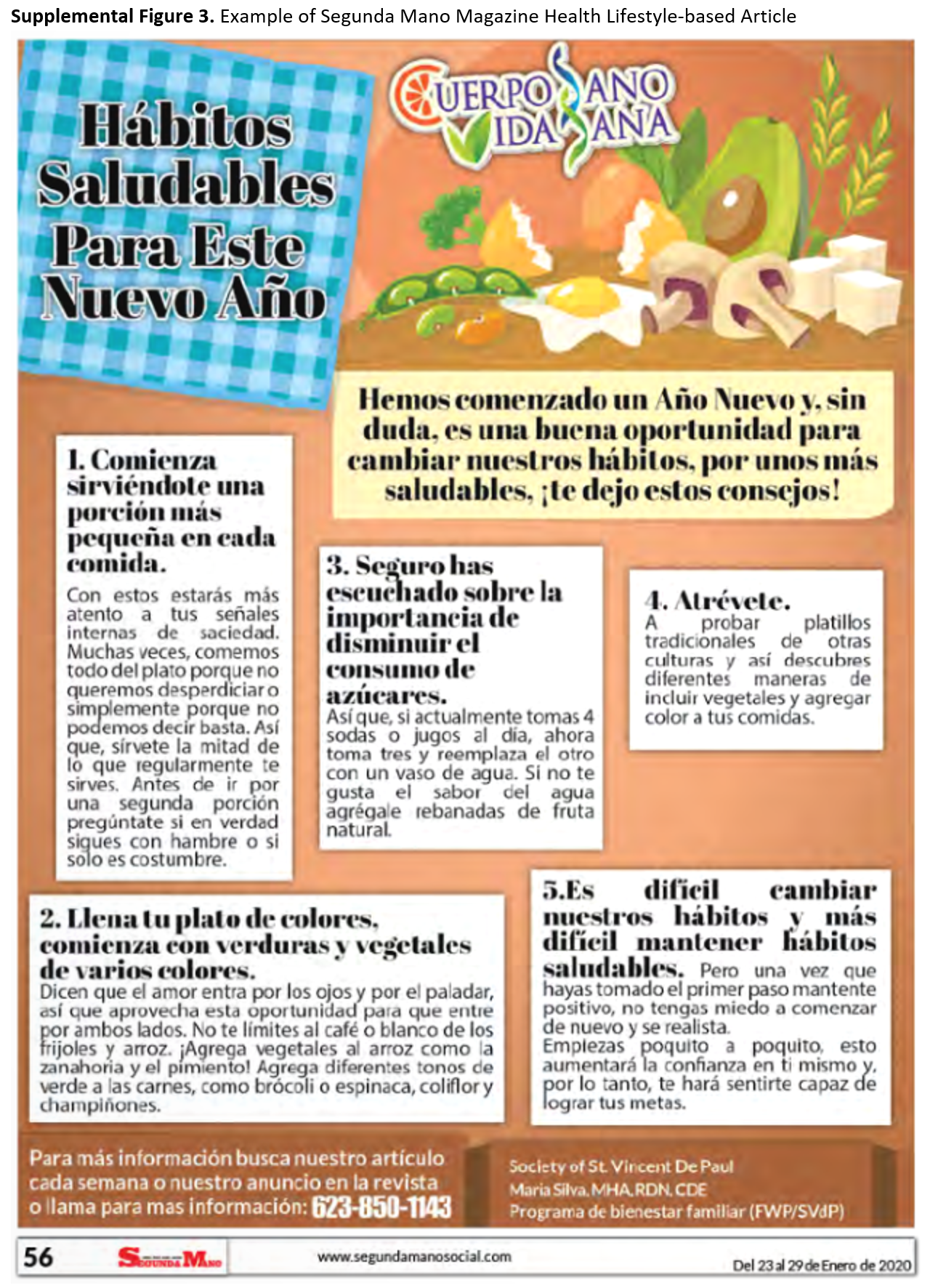

Supplement: Supplementary file 3 — Additional file 3: Supplemental Figure 3. Example of Segunda Mano Magazine Health Lifestyle-based Article. [file 13063_2020_4658_MOESM3_ESM.png]
