# Supplementary material for: Yields and costs of recruitment methods with participant phenotypic characteristics for a diabetes prevention research study in an underrepresented pediatric population
Source: Trials. 2020 Aug 14;21:716. doi: 10.1186/s13063-020-04658-8 (PMC7429699; doi:10.1186/s13063-020-04658-8)
Supplement: Supplementary file 4 — Additional file 4: Supplemental Figure 4. Example Letter Provided to Parent/Guardian of Ineligible Youth. [file 13063_2020_4658_MOESM4_ESM.pdf]

Center for
Health Promotion &
Disease Prevention
ARIZONA STATE UNIVERSITY

May XX, 20XX
Dear: Parent/Guardian Name

Thank you for participating in the ASU Every Little Step Counts study. As you know, we are offering the study to youth whose diabetes test indicate they have Prediabetes and your child's glucose values were **normal** which makes them ineligible for the study. The results from your child's diabetes test and lab visit are enclosed. We recommend that you share these results with your child's primary care provider. These results are for **research purposes only** and do not represent a medical diagnosis. If your child does not have a primary care provider, we have included a list of health clinics that offer low-cost / no-cost health care to the community.

Measure	Your Child's Value	Desirable Value
ALT	XX	5-41 IU/L
AST	XX	10-50 IU/L
Cholesterol	XXX	Less than 160mg/dl
Triglyceride	XX	Less than 89 mg/dl
HDL Cholesterol	XX	More than 46 mg/dl
Non HDL Cholesterol	XXX	Less than 121 mg/dl
LDL Cholesterol	XXX	Less than 109 mg/dl
VLDL Cholesterol	XX	Less than 29 mg/dl
Fasting Glucose	XX	Less than 100 mg/dl
2-Hour Glucose	XXX	Less than 140 mg/dl
Hemoglobin A1c	X.X	Less than 5.7%

Sincerely,

Gabriel Shaibi, PhD
Principal Investigator
Associate Professor
College of Nursing and Health Innovation
602-496-0909
gshaibi@asu.edu

Arizona State University | College of Nursing and Health Innovation
550 North 3rd Street, Phoenix, AZ 85004 | Phone: 602.496.0909 | Fax: 602.496.0886
Email: CHPDP@exchange.asu.edu | nursingandhealth.asu.edu/chpdp

Fecha: XX, 20XX
Estimado(a): Parent/Guardian Name

Agradecemos su participación en el estudio ASU Cada Pasito Cuenta. Como usted sabe, estamos ofreciendo el estudio para aquellos jóvenes a quienes la prueba de Diabetes indica que tienen Prediabetes. Debido a que los resultados de Glucosa de su hijo/a son **normales**, lo hace inelegible para el estudio. Hemos incluido los resultados de la prueba de diabetes y la visita de laboratorio para que los comparta con el médico de atención primaria de su hijo/a. Estos resultados **son para el propósito de la investigación** y no representan un diagnóstico médico. Si su hijo no tiene un médico de atención primaria, hemos incluido una lista de las clínicas de salud que ofrecen servicios de salud a bajo o ningún costo a la comunidad.

**Medidas**	**La Cantidades de su hijo (a)**	**Desirable Value**
ALT	XX	5-41 IU/L
AST	XX	10-50 IU/L
Cholesterol	XXX	Menos de 160mg/dl
Triglyceride	XX	Menos de 89 mg/dl
HDL Cholesterol	XX	Más de 46 mg/dl
Non HDL Cholesterol	XXX	Menos de 121 mg/dl
LDL Cholesterol	XXX	Menos de 109 mg/dl
VLDL Cholesterol	XX	Menos de 29 mg/dl
Fasting Glucose	XX	Menos de 100 mg/dl
2-Hour Glucose	XXX	Menos de 140 mg/dl
Hemoglobin A1c	X.X	Menos de 5.7%

Atentamente,

Gabriel Shaibi, PhD
Principal Investigator
Associate Professor
College of Nursing and Health Innovation
602-496-0909
gshaibi@asu.edu

Center for Health Promotion & Disease Prevention

ARIZONA STATE UNIVERSITY

Clínicas Comunitarias para Participantes de ASU Cada Pasito Cuenta/ Community Medical Resources for ASU ELSC Participants

Mountain Park Health Center

Maryvale - 6601 W Thomas	602-243-7277
Phoenix - 635 E Baseline	602-243-7277
Goodyear -140 N Litchfield Rd.	602-243-7277
Tempe – 1492 S Mill Ave	602-243-7277
Gateway – 3830 E Van Buren	602-243-7277

St. Vincent de Paul Medical Clinic

Phoenix - 420 W Watkins Rd.	602-261-6868

Clínica Adelante

Phoenix- 7725 N 43rd Ave.	1-877-809-5092
Surprise - 15351 W Bell Rd.	1-877-809-5092
Buckeye - 306 E. Monroe	1-877-809-5092
Avondale – 3400 N Dysart Rd	1-877-809-5092
Mesa – 1705 W Main St	1-877-809-5092
Central PHX – 500 W Thomas	1-877-809-5092
Gila Bend – 100 N Gila Bend	1-877-809-5092
Peoria – 15525 N 83rd Ave	1-877-809-5092
Wickenburg – 811 N Tegner St	1-877-809-5092

Wesley Health Center

Phoenix- 1300 S 10th Street	602-257-4338
Phoenix – 1625 N 39th Ave	602-257-4338

Maricopa Integrated Health System

Avondale - 950 East Van Buren	602-344-6800
Chandler - 811 South Hamilton	480-344-6100
El Mirage -12428 West Thunderbird Rd.	623-344-6500
Glendale - 5141 West LaMar	623-344-6700
Guadalupe -5825 East Calle Guadalupe	480-344-6000
Maryvale - 4011 North 51st Avenue	623-344-6900
Mesa - 59 South Hibbert St.	480-344-6200
Seventh Avenue - 1205 South 7th Ave	602-344-6600
Seventh Ave Walk-in - 1201S 7th Ave	602-344-6655
South Central - 33 West Tamarisk	602-344-6400
Sunnyslope - 934 West Hatcher	602-344-6300
Comprehensive Healthcare Center 2525 East Roosevelt Street	602-344-1015
Pendergast – 10550 W Mariposa St	602-344-2520
McDowell – 1101 N Central Ave	602-344-6550
Guadalupe – 5825 E Calle Guadalupe	602-344-6000

Valle del Sol Pediatric Clinic

Phoenix - 3807 N. 7th Street	602-523-9312

Todas las clínicas mencionadas arriba ofrecen atención de bajo o ningún costo. Por favor contacte la clínica de su preferencia para más información o para hacer una cita.

All of the clinics above offer free or reduced cost care. Please contact the individual clinics for more information or to schedule an appointment.

Arizona State University | College of Nursing and Health Innovation
550 North 3rd Street, Phoenix, AZ 85004 | Phone: 602.496.0909 | Fax: 602.496.0886
Email: CHPDP@exchange.asu.edu | nursingandhealth.asu.edu/chpdp
