# Supplementary material for: Yields and costs of recruitment methods with participant phenotypic characteristics for a diabetes prevention research study in an underrepresented pediatric population
Source: Trials. 2020 Aug 14;21:716. doi: 10.1186/s13063-020-04658-8 (PMC7429699; doi:10.1186/s13063-020-04658-8)
Supplement: Supplementary file 5 — Additional file 5: Supplemental Table 1. Participant Reasons for Withdrawing After Randomization. [file 13063_2020_4658_MOESM5_ESM.docx]

| **Supplemental Table 1.** Participant Reasons for Withdrawing After Randomization | | |
| --- | --- | --- |
| **Reason** | **Number** | **Percent** |
| Lack of time/interest | 7 | 46.6% |
| Lost to follow-up | 4 | 26.7% |
| Parent/caregiver schedule conflict | 2 | 13.3% |
| Moved out of state | 1 | 6.7% |
| Parent decline youth blood draw | 1 | 6.7% |
