# Supplementary material for: Yields and costs of recruitment methods with participant phenotypic characteristics for a diabetes prevention research study in an underrepresented pediatric population
Source: Trials. 2020 Aug 14;21:716. doi: 10.1186/s13063-020-04658-8 (PMC7429699; doi:10.1186/s13063-020-04658-8)
Supplement: Supplementary file 6 — Additional file 6: Supplemental Table 2. Baseline Characteristics of Latino Youth Randomized (N = 117) to a Diabetes Prevention Program by Recruitment Site. [file 13063_2020_4658_MOESM6_ESM.docx]

| **Supplemental Table 2.** Baseline Characteristics of Latino Youth Randomized (N=117) to a Diabetes Prevention Program by Recruitment Site | | | | | |
| --- | --- | --- | --- | --- | --- |
| **Source** | **Clinical** | **Community** | **Media** | **Word of Mouth** | **P-Value** |
|  | N = 58 | N = 21 | N = 27 | N = 11 |  |
| Age, years | 13.8±1.3 | 14.0±1.6 | 13.5±1.5 | 13.9±1.5 | 0.65 |
| Sex, % Male (N) | 50.0% (29) | 81.0% (17) | 63.0% (17) | 72.7% (8) | 0.07 |
| Height, cms | 162.3±8.1 | 165.6±9.0 | 162.5±8.9 | 167.9±9.6 | 0.14 |
| Weight, kgs | 91.2±19.7 | 90.8±20.7 | 90.2±21.9 | 97.2±21.0 | 0.80 |
| BMI, kg/m^2^ | 34.1±5.3 | 32.8±4.9 | 33.8±6.3 | 34.2±5.1 | 0.80 |
| BMI Percentile, % | 98.5±1.1 | 98.0±1.6 | 98.5±1.1 | 98.8±0.8 | 0.33 |
| Waist Circumference, cms | 108.5±14.4 | 105.3±12.5 | 105.0±13.5 | 110.1±14.5 | 0.58 |
| Glucose |  |  |  |  |  |
| Fasting, mg/dL | 89.8±6.6 | 89.8±4.6 | 89.6±6.5 | 91.5±10.2 | 0.88 |
| 2-hour, mg/dL | 130.7±19.7 | 118.8±25.2 | 132.7±22.3 | 124.5±23.2 | 0.12 |
| HbA1c, % | 5.6±0.3 | 5.6±0.3 | 5.7±0.2 | 5.6±0.3 | 0.79 |
| Parental T2D History, % Yes (N) | 86.0% (49) | 90.5% (19) | 69.2% (18) | 72.7% (8) | 0.17 |
| Gestational Diabetes Mellitus History, % Yes (N) | 35.1% (20) | 25.0% (5) | 30.8% (8) | 9.1% (1) | 0.36 |
| Data are presented as estimated marginal means and standard error for continuous variables and percentages (N) for categorical variables. Univariate General Linear Models were performed for group comparisons for continuous variables and Chi-square test statistics were performed to compare group differences for categorical variables. Significance was determined at p=0.05. | | | | | |
